# Evaluation of pharmacy-based HIV testing in a high-risk New York City community

**DOI:** 10.1186/1471-2334-14-S2-P4

**Published:** 2014-05-23

**Authors:** S Amesty, N Crawford, V Nandi, A Rivera, C Fuller

**Affiliations:** 1 Columbia University, New York, USA

## Introduction

Injection drug users (IDUs) face limited access to HIV testing and experience delayed HIV diagnoses. New York City (NYC) pharmacies play an important role in HIV prevention among IDUs through the Expanded Syringe Access Program (ESAP), which provides access to sterile syringes without a prescription. We examined in-pharmacy HIV testing among syringe/non-syringe customers in ESAP pharmacies in NYC.

## Materials and methods

Syringe/non-syringe pharmacy customers were recruited in two ESAP community pharmacies in high HIV prevalence neighborhoods to complete a 30-minute survey. In-pharmacy HIV testing was offered to all HIV-negative participants. Descriptive statistics, chi-square and Fisher’s exact tests were performed for categorical outcomes and t-tests for continuous outcomes to determine significant differences in individual characteristics between patrons who accepted in-pharmacy HIV testing compared with those who declined. Stepwise multivariable logistic regression was used to assess the relationship between receiving in-pharmacy HIV testing and customer characteristics identified from bivariate analyses

## Results

Of 327 participants, most were male (57%), black (80%), had ever used hard drugs (88%); 217 (66.4%) were HIV-negative, and 39.6% received in-pharmacy HIV testing. After adjustment, being female (AOR 2.71; 95% CI 1.44-5.10), having multiple sex partners (AOR 1.31; 95% CI 1.08-1.59), having an HIV test more than 12 months ago (AOR 2.90; 95% CI 1.30-6.47), injecting drugs (AOR 2.26; 95% CI 1.04-4.91), and seeing the same regular medical provider (AOR 0.33; 95% CI 0.17-0.67) were associated with receiving in-pharmacy HIV test.

## Conclusions

This study presents evidence for a potential new venue for HIV testing that could provide access for hard-to-reach populations that have limited access to HIV testing. The approach we used was feasible to pharmacists. Given that pharmacies are located everywhere in NYC and have access to new point-of-care and home-based HIV testing technologies, the provision of HIV testing in pharmacies could provide easy access to HIV testing for IDUs as well as other populations.

